# Supplementary material for: Efficacy of the XEN-Implant in Glaucoma and a Meta-Analysis of the Literature
Source: J Clin Med. 2021 Mar 7;10(5):1118. doi: 10.3390/jcm10051118 (PMC7962186; doi:10.3390/jcm10051118)
Supplement: Supplementary file 1 [file jcm-10-01118-s001.pdf]

Supporting materials

Efficacy of the XEN-implant and a meta-analysis of the literature

Contents:

TABLE S1: Summary of the retrieved studies from the systematic review

TABLE S2: Quality assessment of the included non-randomized studies according to the Newcastle-Ottawa Scale

FIGURE S1: Flowchart (according to PRISMA) showing the selection process for inclusion of studies from our searches.

FIGURE S2: Meta-analyses for the effect of XEN-implant on IOP for all specified time-intervalls.

FIGURE S3: Meta-analyses for the effect of XEN-implant on IOP-lowering medication for all specified time-intervalls.

FIGURE S4: Meta-analyses for the postoperative change in intraocular pressure (IOP) and number of IOP-lowering medications for XEN-implant as a standalone and as a combined procedure for all specified time-intervalls. Presented as pooled mean with standard deviation (A) and weighted mean difference (WMD; with corresponding 95% confidence intervals; B).

Table S1. Summary of the retrieved studies from the systematic review.

	Study		Design	*n* (eyes) per Group	Diagnosis	Intervention*	Follow-up months	IOP Reduction mmHg Mean ± s.d. (%)	Needling (%)	Secondary Surgeries (%)	Hypotony (%)	Choroideal Effusion/Detachment (%)	Macular Edema/ Hypotone Maculapathy (%)
1	Smith et al 2019 [29]	R	Case series	68	(P)OAG	XEN + MMC (+ CE *n* = 10)	12	(33)	38.2	10.3	7.4	1.5	NA
2	Reitsamer et al. 2019 [27]	P	Multicenter clinical study	202	(P)OAG	XEN + MMC (+ CE *n* = 79)	24	6.2 ± 4.9 (27.8)	41.1	6.4	2.3	1.8	0.5
3	Heidinger et al. 2019 [20]	R	Cohort study	199	Glaucoma	XEN + MMC	18	(22.7)	22	14	8	0	NA
4	Karimi et al. 2019 [22]	R	Case series	259	Glaucoma	XEN + MMC (+ CE *n* = 72)	12	(26.4)	27	6	23.7	1.5	1.9
5	Widder, et al. 2018 [31]	R	Cohort study	261	Glaucoma	XEN + MMC (+ CE *n* = 49)	12	7.5 ± 10.1 (30.9)	NA	34	NA	3.4	1.7
6	Ozal, et al. 2017 [25]	R	Cohort study	15	(P)OAG, PEG/PEX	XEN (+ CE *n* = 6)	12	(53.6)	0	6.6	0	0	NA
7	Tan, et al. 2017 [30]	R	Cohort study	39	Glaucoma	XEN + MMC (+ CE, *n* = 2)	12	10.4 ± 8.5 (41.8)	17.0	2.6	20.5	NA	NA
8	Grover, et al. 2017 [7]	P	Cohort study	52	(P)OAG, PEG/PEX	XEN + MMC	12	9.1 ± 1.6 (35.6)	32.3	13.8	24.6	3.1	1.5
9	De Gregorio, et al. 2017 [15]	P	Cohort study	41	(P)OAG, PEG/PEX	XEN + MMC +CE	12	9.4 ± 4.4 (41.8)	2.4	2.4	2.4	2.4	NA
10	Galal, et al. 2017 [19]	P	Cohort study	13	(P)OAG	XEN + MMC (+ CE *n* = 10)	12	4 ± 5 (29.4)	30.7	15.4	15.4	15.4	0.
11	Başer et al. 2020 [14]	R	Case series	29	Glaucoma	XEN + MMC (+ CE *n* = 6)	36	(31.6)	27	NA	17	17	NA
12	Fernández et al. 2020 [17]	R	Observational study	63	(P)OAG	XEN + MMC	36	(24.5)	5.4	NA	NA	NA	NA
13	Fea et al. 2020 [16]	P	Multicenter clinical trial	298	GLaucoma	XEN + MMC (+ CE *n* = 56)	12	7.4 ± 7.9 (35.2)	46.2	7	NA	9.4	NA
14	Olgun et al. 2020 [24]	R	Multicenter clinical study	114	Glaucoma	XEN (+ CE *n* = 45)	24	(57.9)	31.5	4.4	0	NA	NA
15	Mansouri et al. 2019 [23]	P	Interventional study	149	Glaucoma	XEN + MMC (+ CE *n* = 109)	24	14.1±3.7 (28.7)	44.4	11.4	2	2.7	0.7
16	Hengerer, et al. 2019 [21]	R	Cohort study	148	(P)OAG	XEN + MMC	12	(53–58)	33.1	7.4	4.7	0	0
17	Gabbay et al. 2019 [18]	R	Cohort study	151	Glaucoma	XEN + MMC (+ CE *n* = 57)	24	(34.6)	36.8	4.6	NA	3	0.6
18	Rauchegger et	R	Observational	60	Glaucoma	XEN + MMC (+	24	(29)	62	16	2.4	2.4	NA

	al. 2020 [26]		study			CE *n* = 23)						
19	Scheres et al. 2020 [28]	R	Comparative study	41	(P)OAG	XEN + MMC (+ CE *n* = 15)	24	(28)	20	14	24	2	NA

* = in brackets the number of eyes from the total study population that underwent a combined procedure; P = prospective; R = retrospective; MMC = Mitomycin C; POAG = primary open angle glaucoma; s.d. = standard deviation; Glaucoma = mixture of different types of glaucoma; PEG = pigment dispersion glaucoma; PEX = pseudoexfoliation glaucoma; OAG = open-angle glaucoma; CE = cataract extraction.

Table S2. Quality assessment of the included non-randomized studies according to the Newcastle-Ottawa Scale.

	Author	Selection	Comparability	Outcome (Cohort)/ Exposure (Case-Control)	Total Score (max.9)
1	Smith et al. 2019 [29]	4	2	2	8
2	Reitsamer et al. 2019 [27]	4	2	2	8
3	Heidinger et al. 2019 [20]	3	2	3	8
4	Karimi et al. 2019 [22]	3	2	2	7
5	Widder, et al. 2018 [31]	4	2	2	8
6	Ozal, et al. 2017 [25]	3	2	2	7
7	Tan, et al. 2017 [30]	3	2	2	7
8	Grover, et al. 2017 [7]	3	2	2	7
9	De Gregorio, et al. 2017 [15]	3	2	3	8
10	Galal, et al. 2017 [19]	3	2	2	7
11	Başer et al. 2020 [14]	4	2	2	8
12	Fernández et al. 2020 [17]	3	2	3	8
13	Fea et al. 2020 [16]	4	2	3	9
14	Olgun et al. 2020 [24]	4	2	3	9
15	Mansouri et al. 2019 [23]	4	2	2	8
16	Hengerer, et al. 2019 [21]	4	2	3	9
17	Gabbay et al. 2019 [18]	4	2	2	8
18	Rauchegger et al. 2020 [26]	3	2	2	7
19	Scheres et al. 2020 [28]	4	2	3	9

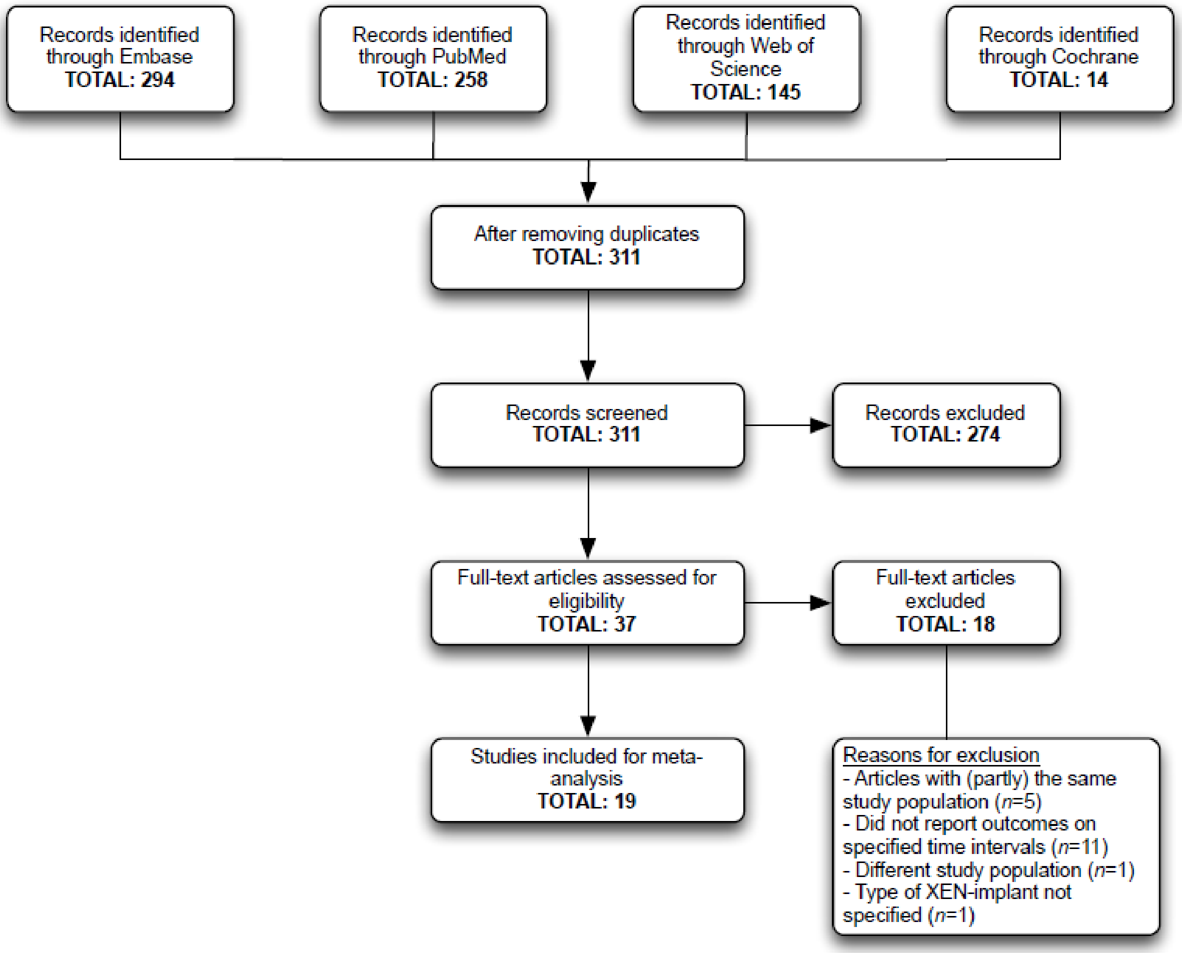

Figure S1. Flowchart (according to PRISMA) showing the selection process for inclusion of studies from our searches*. PRISMA = Preferred Reporting Items for Systematic Reviews and Meta-Analyses; * = Search strings: **Embase:** ('xen'/de OR XEN45/de OR (((xen OR ab-interno) NEAR/3 (stent* OR microstent* OR implant* OR microimplant*)) OR xen45 OR xen-45):ab,ti,kw,dn) OR ((xen OR gelstent* OR gel-stent* OR gelatin*-stent*):ab,ti,kw,dn AND ('glaucoma drainage implant'/de OR 'glaucoma'/exp OR (glaucoma*):ab,ti,kw)) AND [English]/lim NOT [conference abstract]/lim. **Medline ALL Ovid (PubMed):** ((((xen OR ab-interno) ADJ3 (stent* OR microstent* OR implant* OR microimplant*)) OR xen45 OR xen-45).ab,ti,kf.) OR ((xen OR gelstent* OR gel-stent* OR gelatin*-stent*) ab,ti,kf. AND (Glaucoma Drainage Implants / OR Glaucoma / OR (glaucoma*) ab,ti,kf.)) AND english.la. **Web of Science (SCI-EXPANDED & SSCI, 1975-):** TS = (((((xen OR ab-interno) NEAR/2 (stent* OR microstent* OR implant* OR microimplant*)) OR xen45 OR xen-45)) OR ((xen OR gelstent* OR gel-stent* OR gelatin*-stent*) AND ((glaucoma*)))) AND DT = (article) AND LA = (english). **Cochrane CENTRAL register of Trials:** ((((xen OR ab NEXT interno) NEAR/3 (stent* OR microstent* OR implant* OR microimplant*)) OR xen45 OR xen NEXT 45) ab,ti) OR ((xen OR gelstent* OR gel NEXT stent* OR gelatin* NEXT stent*):ab,ti AND ((glaucoma*):ab,ti))

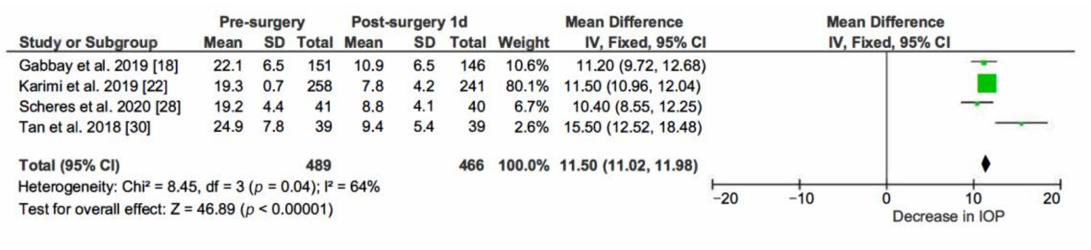
Study or Subgroup | Pre-surgery Mean | SD | Total | Post-surgery 1d Mean | SD | Total | Weight | Mean Difference IV, Fixed, 95% CI
Gabbay et al. 2019 [18] 22.1 6.5 151 10.9 6.5 146 10.6% 11.20 (9.72, 12.68)
Karimi et al. 2019 [22] 19.3 0.7 258 7.8 4.2 241 80.1% 11.50 (10.96, 12.04)
Scheres et al. 2020 [28] 19.2 4.4 41 8.8 4.1 40 6.7% 10.40 (8.55, 12.25)
Tan et al. 2018 [30] 24.9 7.8 39 9.4 5.4 39 2.6% 15.50 (12.52, 18.48)
Total (95% CI) 489 466 100.0% 11.50 (11.02, 11.98)
Heterogeneity: Chi² = 8.45, df = 3 (p = 0.04); I² = 64%
Test for overall effect: Z = 46.89 (p < 0.00001)
Mean Difference IV, Fixed, 95% CI
−20 −10 0 10 20
Decrease in IOP

(**A**)

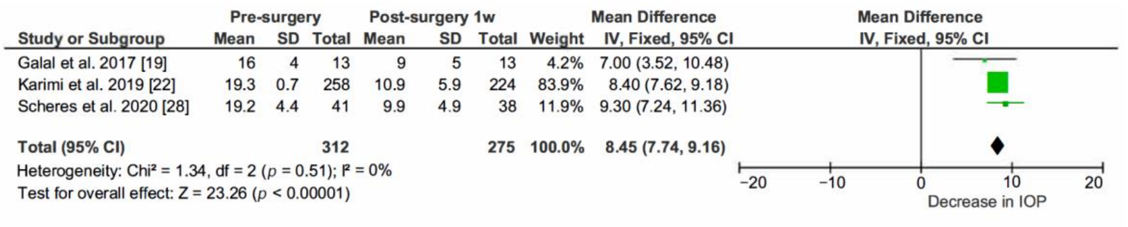
Study or Subgroup | Pre-surgery Mean | SD | Total | Post-surgery 1w Mean | SD | Total | Weight | Mean Difference IV, Fixed, 95% CI
Galal et al. 2017 [19] 16 4 13 9 5 13 4.2% 7.00 (3.52, 10.48)
Karimi et al. 2019 [22] 19.3 0.7 258 10.9 5.9 224 83.9% 8.40 (7.62, 9.18)
Scheres et al. 2020 [28] 19.2 4.4 41 9.9 4.9 38 11.9% 9.30 (7.24, 11.36)
Total (95% CI) 312 275 100.0% 8.45 (7.74, 9.16)
Heterogeneity: Chi² = 1.34, df = 2 (p = 0.51); I² = 0%
Test for overall effect: Z = 23.26 (p < 0.00001)
Mean Difference IV, Fixed, 95% CI
−20 −10 0 10 20
Decrease in IOP

(**B**)

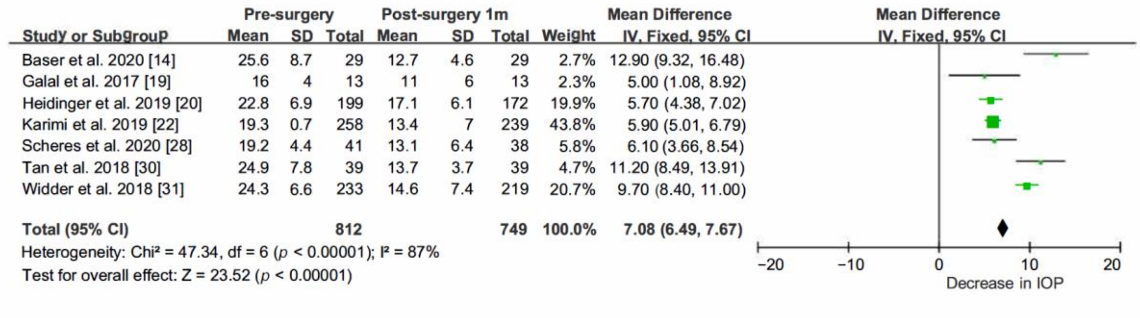
Study or Subgroup | Pre-surgery Mean | SD | Total | Post-surgery 1m Mean | SD | Total | Weight | Mean Difference IV, Fixed, 95% CI
Baser et al. 2020 [14] 25.6 8.7 29 12.7 4.6 29 2.7% 12.90 (9.32, 16.48)
Galal et al. 2017 [19] 16 4 13 11 6 13 2.3% 5.00 (1.08, 8.92)
Heidinger et al. 2019 [20] 22.8 6.9 199 17.1 6.1 172 19.9% 5.70 (4.38, 7.02)
Karimi et al. 2019 [22] 19.3 0.7 258 13.4 7 239 43.8% 5.90 (5.01, 6.79)
Scheres et al. 2020 [28] 19.2 4.4 41 13.1 6.4 38 5.8% 6.10 (3.66, 8.54)
Tan et al. 2018 [30] 24.9 7.8 39 13.7 3.7 39 4.7% 11.20 (8.49, 13.91)
Widder et al. 2018 [31] 24.3 6.6 233 14.6 7.4 219 20.7% 9.70 (8.40, 11.00)
Total (95% CI) 812 749 100.0% 7.08 (6.49, 7.67)
Heterogeneity: Chi² = 47.34, df = 6 (p < 0.00001); I² = 87%
Test for overall effect: Z = 23.52 (p < 0.00001)
Mean Difference IV, Fixed, 95% CI
−20 −10 0 10 20
Decrease in IOP

(**C**)

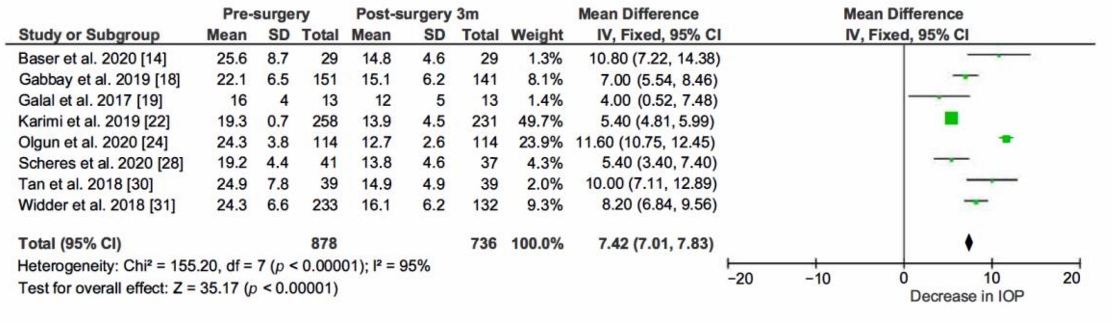
Study or Subgroup | Pre-surgery Mean | SD | Total | Post-surgery 3m Mean | SD | Total | Weight | Mean Difference IV, Fixed, 95% CI
Baser et al. 2020 [14] 25.6 8.7 29 14.8 4.6 29 1.3% 10.80 (7.22, 14.38)
Gabbay et al. 2019 [18] 22.1 6.5 151 15.1 6.2 141 8.1% 7.00 (5.54, 8.46)
Galal et al. 2017 [19] 16 4 13 12 5 13 1.4% 4.00 (0.52, 7.48)
Karimi et al. 2019 [22] 19.3 0.7 258 13.9 4.5 231 49.7% 5.40 (4.81, 5.99)
Olgun et al. 2020 [24] 24.3 3.8 114 12.7 2.6 114 23.9% 11.60 (10.75, 12.45)
Scheres et al. 2020 [28] 19.2 4.4 41 13.8 4.6 37 4.3% 5.40 (3.40, 7.40)
Tan et al. 2018 [30] 24.9 7.8 39 14.9 4.9 39 2.0% 10.00 (7.11, 12.89)
Widder et al. 2018 [31] 24.3 6.6 233 16.1 6.2 132 9.3% 8.20 (6.84, 9.56)
Total (95% CI) 878 736 100.0% 7.42 (7.01, 7.83)
Heterogeneity: Chi² = 155.20, df = 7 (p < 0.00001); I² = 95%
Test for overall effect: Z = 35.17 (p < 0.00001)
Mean Difference IV, Fixed, 95% CI
−20 −10 0 10 20
Decrease in IOP

(**D**)

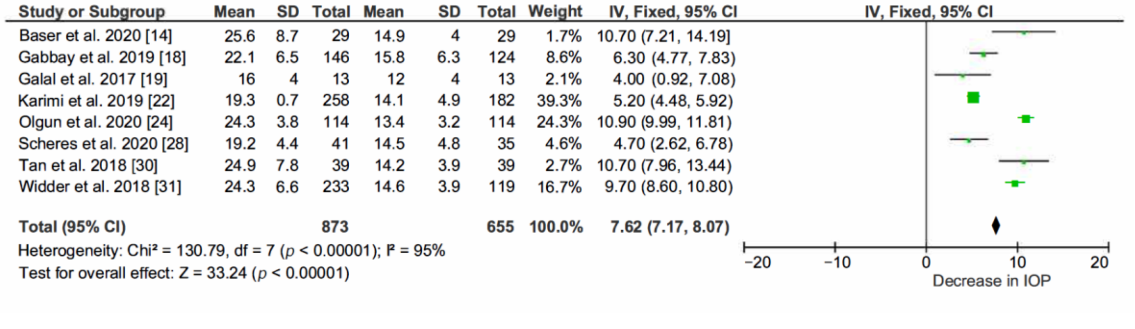
Study or Subgroup | Pre-surgery Mean | SD | Total | Post-surgery 6m Mean | SD | Total | Weight | Mean Difference IV, Fixed, 95% CI
Baser et al. 2020 [14] 25.6 8.7 29 14.9 4 29 1.7% 10.70 (7.21, 14.19)
Gabbay et al. 2019 [18] 22.1 6.5 146 15.8 6.3 124 8.6% 6.30 (4.77, 7.83)
Galal et al. 2017 [19] 16 4 13 12 4 13 2.1% 4.00 (0.92, 7.08)
Karimi et al. 2019 [22] 19.3 0.7 258 14.1 4.9 182 39.3% 5.20 (4.48, 5.92)
Olgun et al. 2020 [24] 24.3 3.8 114 13.4 3.2 114 24.3% 10.90 (9.99, 11.81)
Scheres et al. 2020 [28] 19.2 4.4 41 14.5 4.8 35 4.6% 4.70 (2.62, 6.78)
Tan et al. 2018 [30] 24.9 7.8 39 14.2 3.9 39 2.7% 10.70 (7.96, 13.44)
Widder et al. 2018 [31] 24.3 6.6 233 14.6 3.9 119 16.7% 9.70 (8.60, 10.80)
Total (95% CI) 873 655 100.0% 7.62 (7.17, 8.07)
Heterogeneity: Chi² = 130.79, df = 7 (p < 0.00001); I² = 95%
Test for overall effect: Z = 33.24 (p < 0.00001)
Mean Difference IV, Fixed, 95% CI
−20 −10 0 10 20
Decrease in IOP

(**E**)

Study or Subgroup	Pre-surgery Mean	Pre-surgery SD	Pre-surgery Total	Post-surgery 1y Mean	Post-surgery 1y SD	Post-surgery 1y Total	Weight	Mean Difference IV, Fixed, 95% CI
Baser et al. 2020 [14]	25.6	8.7	29	15.8	4.5	24	0.7%	9.80 (6.16, 13.44)
De Gregorio et al. 2018 [15]	22.5	3.7	41	13.1	2.4	40	5.2%	9.40 (8.05, 10.75)
Fea et al. 2020 [16]	23.9	7	298	15.5	3.9	171	9.9%	8.40 (7.41, 9.39)
Fernández et al. 2020 [17]	18.2	5	93	14.2	2.1	93	7.9%	4.00 (2.90, 5.10)
Gabbay et al. 2019 [18]	22.1	6.5	151	15.4	5.9	109	4.2%	6.70 (5.18, 8.22)
Galal et al. 2017 [19]	16	4	13	12	3	13	1.3%	4.00 (1.28, 6.72)
Grover et al. 2017 [7]	25.1	3.7	65	15.9	5.2	52	3.4%	9.20 (7.52, 10.88)
Heidinger et al. 2019 [20]	22.8	6.9	199	17.1	5.9	89	4.0%	5.70 (4.14, 7.26)
Hengerer et al. 2019 [21]	32.9	9.7	148	14.3	4.5	148	3.2%	18.60 (16.88, 20.32)
Karimi et al. 2019 [22]	19.3	0.7	258	14.2	4.4	89	11.4%	5.10 (4.18, 6.02)
Mansouri et al. 2019 [23]	20	7.1	149	14.3	4.4	149	5.3%	5.70 (4.36, 7.04)
Olgun et al. 2020 [24]	24.3	3.8	114	13.5	2.3	114	14.4%	10.80 (9.98, 11.62)
Rauchegger et al. 2020 [26]	23.4	7.9	60	14.6	3.6	30	1.7%	8.80 (6.42, 11.18)
Reitsamer et al. 2019 [27]	21.4	3.6	202	14.9	4.5	178	14.0%	6.50 (5.67, 7.33)
Scheres et al. 2020 [28]	19.2	4.4	41	13.3	2.9	30	3.3%	5.90 (4.20, 7.60)
Smith et al. 2019 [29]	22.1	6.4	68	14.8	5.1	68	2.5%	7.30 (5.35, 9.25)
Tan et al. 2018 [30]	24.9	7.8	39	14.5	3.4	39	1.3%	10.40 (7.73, 13.07)
Widder et al. 2018 [31]	24.3	6.6	233	13.9	3.8	65	6.1%	10.40 (9.15, 11.65)
**Total (95% CI)**			**2201**			**1501**	**100.0%**	**7.84 (7.53, 8.15)**

Heterogeneity: Chi² = 353.75, df = 17 (p < 0.00001); I² = 95%
Test for overall effect: Z = 49.61 (p < 0.00001)

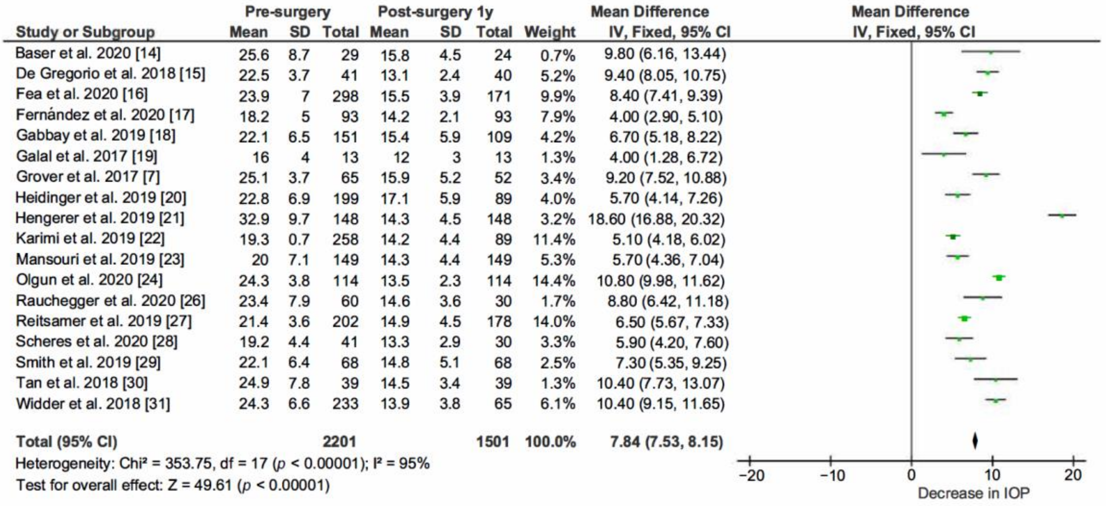

(**F**)

Study or Subgroup	Pre-surgery Mean	Pre-surgery SD	Pre-surgery Total	Post-surgery 18m Mean	Post-surgery 18m SD	Post-surgery 18m Total	Weight	Mean Difference IV, Fixed, 95% CI
Baser et al. 2020 [14]	25.6	8.7	29	15.8	4.5	24	2.1%	9.80 (6.16, 13.44)
Gabbay et al. 2019 [18]	22.1	6.5	151	15.8	5.8	76	10.1%	6.30 (4.63, 7.97)
Heidinger et al. 2019 [20]	22.8	6.9	199	16.4	3.8	43	12.7%	6.40 (4.91, 7.89)
Karimi et al. 2019 [22]	19.3	0.7	258	13.5	3.3	32	21.4%	5.80 (4.65, 6.95)
Olgun et al. 2020 [24]	24.3	3.8	114	13.9	2.5	114	40.3%	10.40 (9.57, 11.23)
Scheres et al. 2020 [28]	19.2	4.4	41	13.3	2.9	30	9.7%	5.90 (4.20, 7.60)
Widder et al. 2018 [31]	24.3	6.6	233	14.5	4.9	13	3.6%	9.80 (7.00, 12.60)
**Total (95% CI)**			**1025**			**332**	**100.0%**	**8.02 (7.49, 8.55)**

Heterogeneity: Chi² = 62.72, df = 6 (p < 0.00001); I² = 90%
Test for overall effect: Z = 29.65 (p < 0.00001)

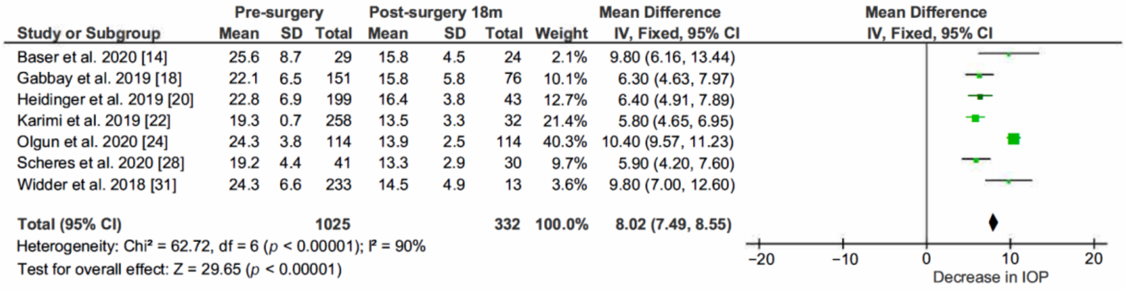

Study or Subgroup	Pre-surgery Mean	Pre-surgery SD	Pre-surgery Total	Post-surgery 2y Mean	Post-surgery 2y SD	Post-surgery 2y Total	Weight	Mean Difference IV, Fixed, 95% CI
Baser et al. 2020 [14]	25.6	8.7	29	15.6	3.2	15	1.5%	10.00 (6.44, 13.56)
Fernández et al. 2020 [17]	18.2	5	93	14.5	2.2	45	12.9%	3.70 (2.50, 4.90)
Gabbay et al. 2019 [18]	22.1	6.5	151	14.5	3.3	48	9.6%	7.60 (6.20, 9.00)
Mansouri et al. 2019 [23]	20	7.1	149	14.1	3.7	129	11.0%	5.90 (4.59, 7.21)
Olgun et al. 2020 [24]	24.3	3.8	114	13.8	2.1	114	29.4%	10.50 (9.70, 11.30)
Rauchegger et al. 2020 [26]	23.4	7.9	60	14.8	4.4	28	2.8%	8.60 (6.02, 11.18)
Reitsamer et al. 2019 [27]	21.4	3.6	202	15.2	4.2	161	28.0%	6.20 (5.38, 7.02)
Scheres et al. 2020 [28]	19.2	4.4	41	13.8	3.8	26	4.7%	5.40 (3.41, 7.39)
**Total (95% CI)**			**839**			**566**	**100.0%**	**7.33 (6.90, 7.76)**

Heterogeneity: Chi² = 114.60, df = 7 (p < 0.00001); I² = 94%
Test for overall effect: Z = 33.22 (p < 0.00001)

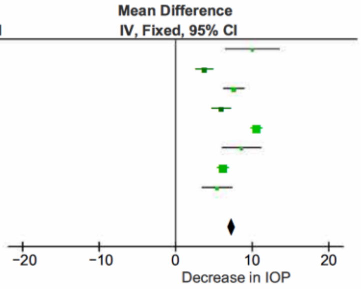

(**G**)

(**H**)

Figure S2. Meta-analyses for the effect of XEN-implant on IOP for all specified time-intervalls.*. * = Presented as mean decrease in IOP at 1 day (**A**), 1 week (**B**), 1 month (**C**), 3 months (**D**), 6 months (**E**), 1 year (**F**), 1.5 years (**G**) and 2 years (**H**) follow-up. Black diamonds indicate the overall weighted mean difference (WMD). The size of the green box is inversely proportional to the variance. Horizontal lines indicate 95% confidence interval (CI). The dashed vertical line in each panel shows the value for no effect (WMD = 0) compared to the pre-surgery IOP. IOP = intraocular pressure; CI = confidence interval; SD = standard deviation; IV = inverse variance.

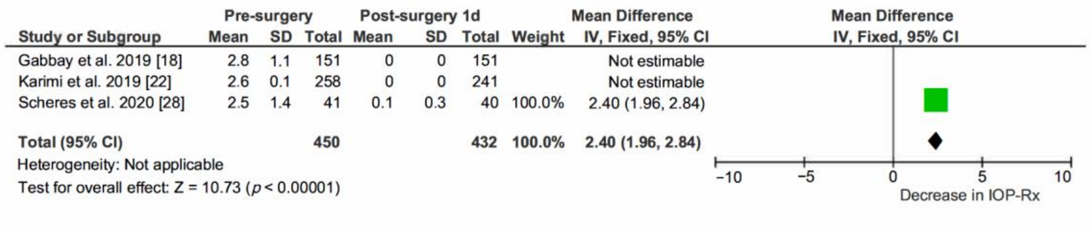
Pre-surgery
Post-surgery 1d
Mean Difference
Mean Difference
Study or Subgroup Mean SD Total Mean SD Total Weight IV, Fixed, 95% CI
IV, Fixed, 95% CI
Gabbay et al. 2019 [18] 2.8 1.1 151 0 0 151 Not estimable
Karimi et al. 2019 [22] 2.6 0.1 258 0 0 241 Not estimable
Scheres et al. 2020 [28] 2.5 1.4 41 0.1 0.3 40 100.0% 2.40 (1.96, 2.84)
Total (95% CI) 450 432 100.0% 2.40 (1.96, 2.84)
Heterogeneity: Not applicable
Test for overall effect: Z = 10.73 (p < 0.00001)
−10 −5 0 5 10
Decrease in IOP-Rx

(**A**)

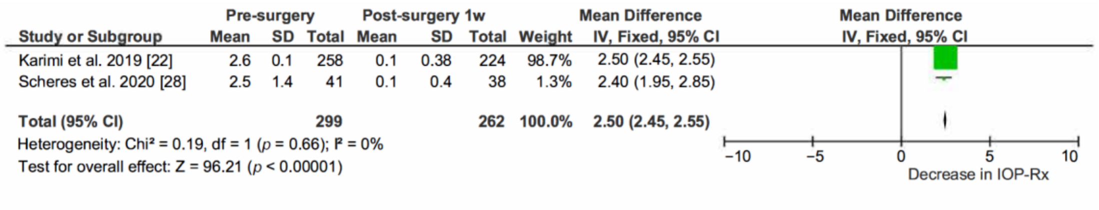
Pre-surgery
Post-surgery 1w
Mean Difference
Mean Difference
Study or Subgroup Mean SD Total Mean SD Total Weight IV, Fixed, 95% CI
IV, Fixed, 95% CI
Karimi et al. 2019 [22] 2.6 0.1 258 0.1 0.38 224 98.7% 2.50 (2.45, 2.55)
Scheres et al. 2020 [28] 2.5 1.4 41 0.1 0.4 38 1.3% 2.40 (1.95, 2.85)
Total (95% CI) 299 262 100.0% 2.50 (2.45, 2.55)
Heterogeneity: Chi² = 0.19, df = 1 (p = 0.66); I² = 0%
Test for overall effect: Z = 96.21 (p < 0.00001)
−10 −5 0 5 10
Decrease in IOP-Rx

(**B**)

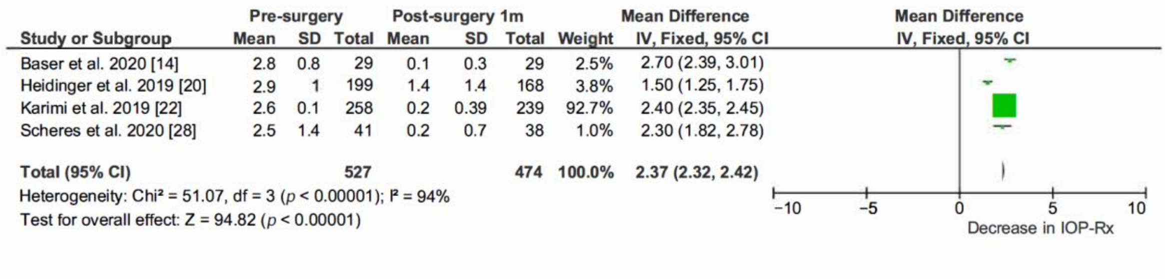
Pre-surgery
Post-surgery 1m
Mean Difference
Mean Difference
Study or Subgroup Mean SD Total Mean SD Total Weight IV, Fixed, 95% CI
IV, Fixed, 95% CI
Baser et al. 2020 [14] 2.8 0.8 29 0.1 0.3 29 2.5% 2.70 (2.39, 3.01)
Heidinger et al. 2019 [20] 2.9 1 199 1.4 1.4 168 3.8% 1.50 (1.25, 1.75)
Karimi et al. 2019 [22] 2.6 0.1 258 0.2 0.39 239 92.7% 2.40 (2.35, 2.45)
Scheres et al. 2020 [28] 2.5 1.4 41 0.2 0.7 38 1.0% 2.30 (1.82, 2.78)
Total (95% CI) 527 474 100.0% 2.37 (2.32, 2.42)
Heterogeneity: Chi² = 51.07, df = 3 (p < 0.00001); I² = 94%
Test for overall effect: Z = 94.82 (p < 0.00001)
−10 −5 0 5 10
Decrease in IOP-Rx

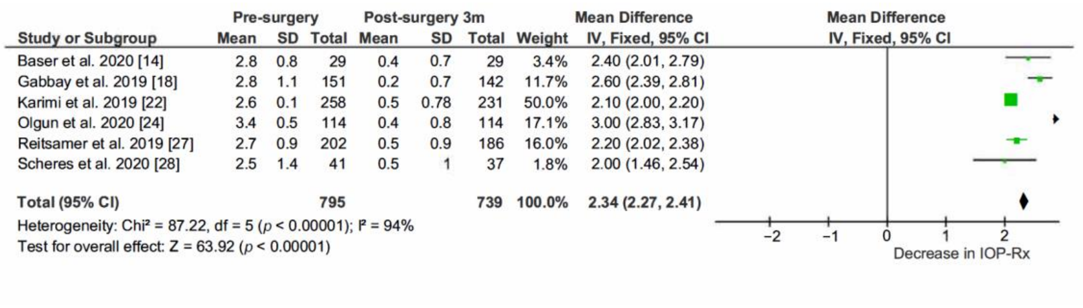
Pre-surgery
Post-surgery 3m
Mean Difference
Mean Difference
Study or Subgroup Mean SD Total Mean SD Total Weight IV, Fixed, 95% CI
IV, Fixed, 95% CI
Baser et al. 2020 [14] 2.8 0.8 29 0.4 0.7 29 3.4% 2.40 (2.01, 2.79)
Gabbay et al. 2019 [18] 2.8 1.1 151 0.2 0.7 142 11.7% 2.60 (2.39, 2.81)
Karimi et al. 2019 [22] 2.6 0.1 258 0.5 0.78 231 50.0% 2.10 (2.00, 2.20)
Olgun et al. 2020 [24] 3.4 0.5 114 0.4 0.8 114 17.1% 3.00 (2.83, 3.17)
Reitsamer et al. 2019 [27] 2.7 0.9 202 0.5 0.9 186 16.0% 2.20 (2.02, 2.38)
Scheres et al. 2020 [28] 2.5 1.4 41 0.5 1 37 1.8% 2.00 (1.46, 2.54)
Total (95% CI) 795 739 100.0% 2.34 (2.27, 2.41)
Heterogeneity: Chi² = 87.22, df = 5 (p < 0.00001); I² = 94%
Test for overall effect: Z = 63.92 (p < 0.00001)
−2 −1 0 1 2
Decrease in IOP-Rx

(**C**)

(**D**)

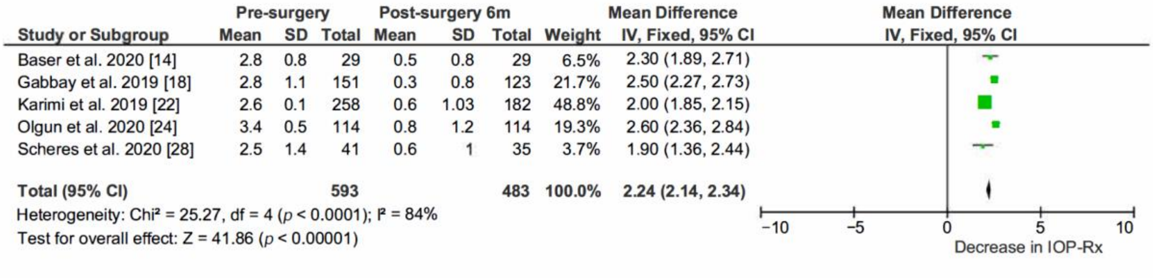
Pre-surgery
Post-surgery 6m
Mean Difference
Mean Difference
Study or Subgroup Mean SD Total Mean SD Total Weight IV, Fixed, 95% CI
IV, Fixed, 95% CI
Baser et al. 2020 [14] 2.8 0.8 29 0.5 0.8 29 6.5% 2.30 (1.89, 2.71)
Gabbay et al. 2019 [18] 2.8 1.1 151 0.3 0.8 123 21.7% 2.50 (2.27, 2.73)
Karimi et al. 2019 [22] 2.6 0.1 258 0.6 1.03 182 48.8% 2.00 (1.85, 2.15)
Olgun et al. 2020 [24] 3.4 0.5 114 0.8 1.2 114 19.3% 2.60 (2.36, 2.84)
Scheres et al. 2020 [28] 2.5 1.4 41 0.6 1 35 3.7% 1.90 (1.36, 2.44)
Total (95% CI) 593 483 100.0% 2.24 (2.14, 2.34)
Heterogeneity: Chi² = 25.27, df = 4 (p < 0.0001); I² = 84%
Test for overall effect: Z = 41.86 (p < 0.00001)
−10 −5 0 5 10
Decrease in IOP-Rx

(**E**)

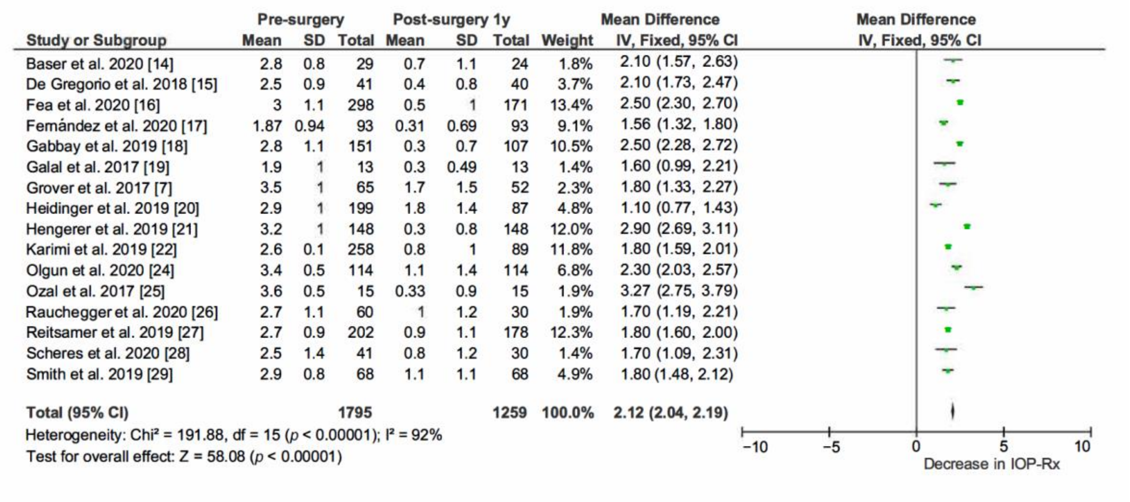

(**F**)

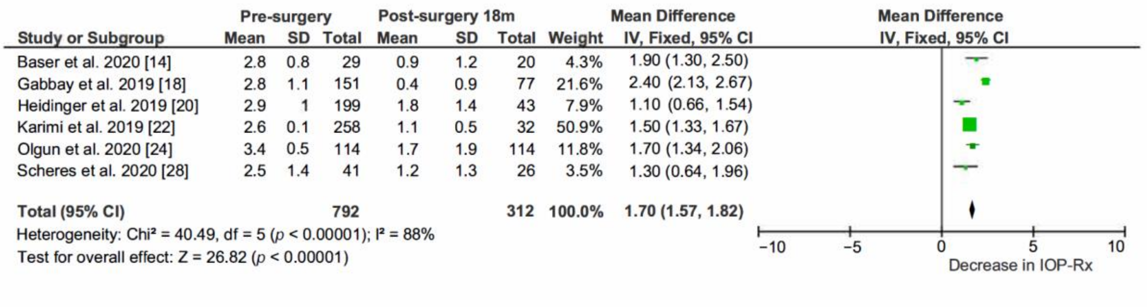

(**G**)

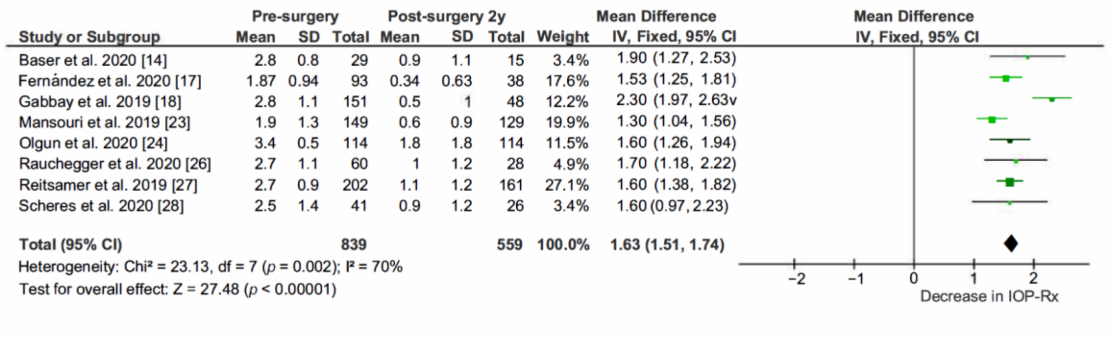

(**H**)

Figure S3. Meta-analyses for the effect of XEN-implant on IOP-lowering medication for all specified time-intervalls.*. * = Presented as mean decrease in IOP-lowering medication at 1 day (**A**), 1 week (**B**), 1 month (**C**), 3 months (**D**), 6 months (**E**), 1 year (**F**), 1.5 years (**G**) and 2 years (**H**) follow-up. Black diamonds indicate the overall weighted mean difference (WMD). The size of the green box is inversely proportional to the variance. Horizontal lines indicate 95% confidence interval (CI). The dashed vertical line in each panel shows the value for no effect (WMD = 0) compared to the pre-surgery IOP-lowering medication. IOP = intraocular pressure; CI = confidence interval; SD = standard deviation; IV = inverse variance.

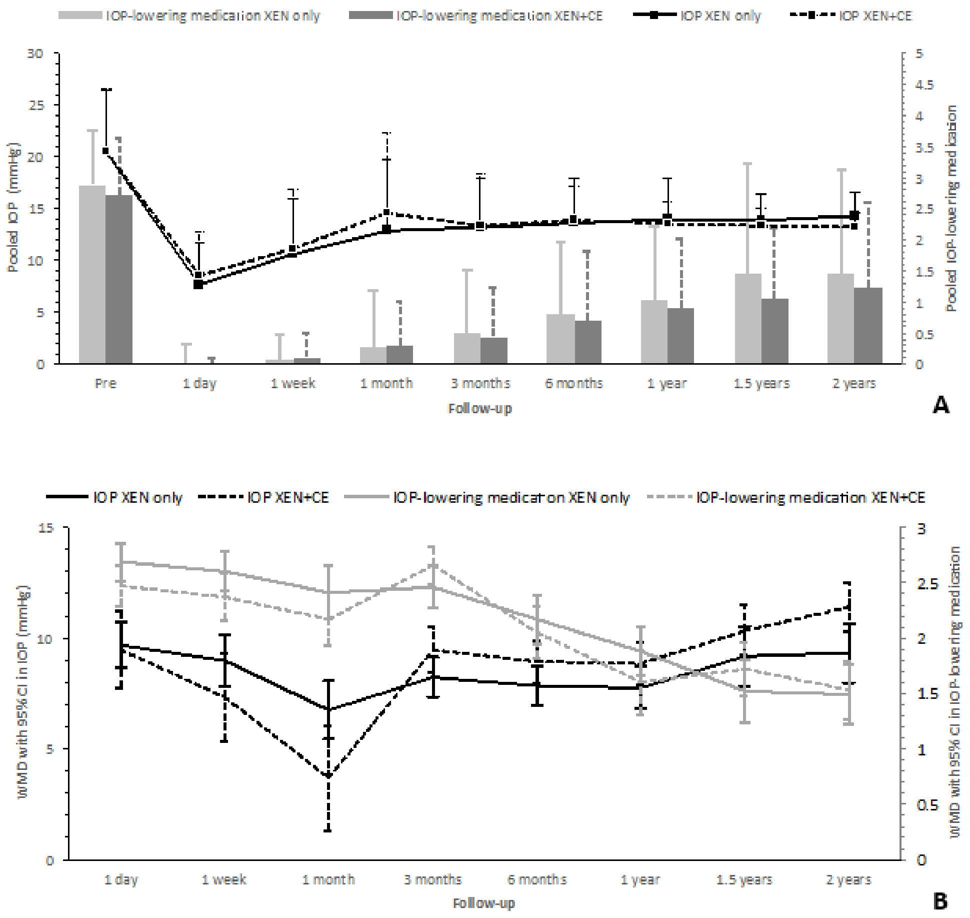

XEN		Pre	1 day	1 week	1 month	3 months	6 months	1 year	1.5 years	2 years
standalone	N (IOP)	263	205	191	204	238	209	141	67	67
combined	N (IOP)	132	76	71	73	126	104	74	55	55
standalone	N (IOP-lowering medication)	191	140	132	129	175	178	167	151	153
combined	N (IOP-lowering medication)	148	102	99	96	142	139	137	134	130

Figure S4. Meta-analyses for the postoperative change in intraocular pressure (IOP) and number of IOP-lowering medications for XEN-implant as a standalone and as a combined procedure for all specified time-intervalls. Presented as pooled mean with standard deviation (**A**) and weighted mean difference (WMD) with corresponding 95% confidence intervals (**B**). CE = cataract extraction (i.e., combined procedure).
